# HIV Treatment as Prevention: Considerations in the Design, Conduct, and Analysis of Cluster Randomized Controlled Trials of Combination HIV Prevention

**DOI:** 10.1371/journal.pmed.1001250

**Published:** 2012-07-10

**Authors:** Marie-Claude Boily, Benoît Mâsse, Ramzi Alsallaq, Nancy S. Padian, Jeffrey W. Eaton, Juan F. Vesga, Timothy B. Hallett

**Affiliations:** 1 Department of Infectious Disease Epidemiology, School of Public Health, Imperial College London, London, United Kingdom; 2 CHU Sainte-Justine Research Centre, University of Montreal, Montreal, Quebec, Canada; 3 College of Nursing Global, New York University, New York, New York, United States of America; 4 Office of the US Global AIDS Coordinator, US Department of State, Washington, District of Columbia, United States of America; 5 Department of Epidemiology, University of California, Berkeley, California, United States of America; Duke University Medical Center, United States of America

## Abstract

The rigorous evaluation of the impact of combination HIV prevention packages at the population level will be critical for the future of HIV prevention. In this review, we discuss important considerations for the design and interpretation of cluster randomized controlled trials (C-RCTs) of combination prevention interventions. We focus on three large C-RCTs that will start soon and are designed to test the hypothesis that combination prevention packages, including expanded access to antiretroviral therapy, can substantially reduce HIV incidence. Using a general framework to integrate mathematical modelling analysis into the design, conduct, and analysis of C-RCTs will complement traditional statistical analyses and strengthen the evaluation of the interventions. Importantly, even with combination interventions, it may be challenging to substantially reduce HIV incidence over the 2- to 3-y duration of a C-RCT, unless interventions are scaled up rapidly and key populations are reached. Thus, we propose the innovative use of mathematical modelling to conduct interim analyses, when interim HIV incidence data are not available, to allow the ongoing trials to be modified or adapted to reduce the likelihood of inconclusive outcomes. The preplanned, interactive use of mathematical models during C-RCTs will also provide a valuable opportunity to validate and refine model projections.

## Rationale for Cluster Randomized Controlled Trials

Significant progress has been achieved in developing, implementing, and scaling- up safe and effective biomedical and behavioural HIV interventions such as promoting condom use, male circumcision (MC), and the use of antiretroviral drugs for treatment and for the prevention of mother-to-child and heterosexual transmission [1]. Other interventions, such as oral or topical pre-exposure prophylaxis, are in the late stages of clinical evaluation [2]. Considered alone, each intervention provides only partial protection or requires high levels of individual adherence. The combination of several prevention interventions could achieve substantial reductions in incidence even if coverage and adherence to each intervention is suboptimal. The combination approach is widely seen as the most promising way to control the HIV epidemic, especially in highly endemic countries [3],[4]. However, the potential population-level effectiveness or impact of combination prevention packages is difficult to predict and needs to be rigorously evaluated in real world settings.

The impact of an intervention at the population level can be very different from its observed efficacy in clinical trials for many reasons, including differences in implementation (e.g., speed and quality of scale-up), target population (e.g., universal, or key subpopulations), and in individual-level factors (e.g., adherence, uptake, sexual behaviour disinhibition) [5]–[7]. In addition, the level of indirect or herd effects on those not receiving the intervention as a result of the decreasing prevalence of infectious individuals over time is not captured in individual-based randomized controlled trials (I-RCTs) and may differ between interventions [5]–[7]. Cluster randomized controlled trials (C-RCTs; also called community-based RCTs) are trials in which whole communities, or clusters of individuals, are randomly allocated to receive either the intervention or the control condition [5],[8]. C-RCTs can be used to measure the population-level impact of an intervention [5],[8]. Typically, the intervention is implemented across the trial communities, but the population-level impact is assessed by measuring the incidence rate among a cohort of individuals in the intervention group compared with a cohort in the control group.

Three large C-RCTs commissioned by the US President's Emergency Plan for AIDS Relief (PEPFAR) to measure the impact of combination prevention packages (including expanded access to antiretroviral therapy [ART]) on HIV incidence in different populations will start shortly (Table 1) [9]–[11]. The different trial intervention packages focus on the scale-up of ART (i) initiated at different CD4 levels in Zambia and South Africa, (ii) prioritising those with the highest viral loads in Botswana, and (iii) in combination with other interventions in Tanzania.

**Table 1:** Main characteristics of cluster randomized controlled trials for combination prevention of HIV transmission commissioned by PEPFAR.

Study	CDC/HSPH^a^	JHU/USAID	PopART (HPTN 071)
**Site**	Botswana	Iringa, Tanzania	Zambia+South Africa (Western Cape)
**Number of arms**	2	2	3
**Intervention arm(s)**	A: Enhanced HIV testing (including mobile and home-based testing), active linkage to care and treatment; enhanced MC; ART for all HIV-infected persons with CD4<350 cells/µl or with HIV-1 RNA>10,000 copies/ml; and point-of-care CD4 testing in antenatal clinics with universal HAART in pregnancy started by 28 wk gestation, as well as HIV retesting at delivery among women HIV-negative in second trimester or earlier	A: Treatment by CD4<350 cells/µl; active scale-up and linkage to MC; cash transfer for young women; targeted outreach to the most at-risk populations (including female sex workers); social and behaviour change communication	A: Universal community home-based testing; active linkage of HIV-positive individuals to care and immediate ART according to national guidelines and/or MC. B: Same as A but ART at CD4<350 cells/µl
**Control arm**	B: Standard of care^b^	B: Standard of care^c^	C: Enhanced standard of care^d^
**Design**	Pair matched	Stratified	Triplet matched
**Number of randomized clusters**			
Total	30	24	24 (South Africa: 9, Zambia: 15)
Per arm	15	12	8
**Average size of randomized cluster**	5,800	8,000–10,000 (∼55%>15 y)	50,000 (25,000>18 y)
**Overall cohort followed up**			
Age eligibility	16–64 y	15–39 y	18–44 y
Size per cluster	∼500 adults per cluster	∼500 adults per cluster	∼2,500 adults per cluster
Total size	15,000	12,000	60,000
**Primary Outcome**	HIV incidence^e^	HIV incidence^e^	HIV incidence^e^
**Follow-up duration**	3–4 y	2 y	2 y
**HIV incidence assumption**	∼1.5 per 100 person-years	1 .0–1.5 per 100 person-years	1.0–1.5 per 100 person-years
**Anticipated HIV prevalence at baseline**	25%	10%–15%	15%
**Target reduction in incidence**	In arm A versus B: ∼50%	In arm A versus B: ∼40% (35%–50%)	In arm A versus C: −50% to 60%; in arm B versus C: −25% to 30%
**Stages when modelling is currently planned**	Start	Start, interim, final	Start, final
**Status**	Planning	Pre-trial	Pre-trial

Data as of 15 March 2012.

a The design of the intervention and plan of analysis for this trial are still being finalised.

b Standard of care is ART for HIV-positive individuals with CD4<350 cells/µl or AIDS.

c Standard of care is standard referral to MC and ART according to Tanzania guidelines (this will soon change from CD4<200 cells/µl to CD4<350 cells/µl, initially focusing on HIV-positive people with tuberculosis and pregnant women).

d Standard of care is no home-based testing or home-based visit to facilitate linkage to ART. ART given according to country guidelines; standard referral to MC.

e Cumulative HIV incidence measured over the trial duration.

CDC/HSPH, US Centers for Disease Control and Prevention/Harvard School of Public Health; HAART, highly active ART; JHU/USAID, Johns Hopkins University/United States Agency of International Development; PopART (HPTN 071), HIV Prevention Trials Network.

In a context in which resources generally are becoming increasingly scarce, obtaining valid answers from these trials will be critical for the future of HIV prevention. Positive results showing large reductions in HIV incidence could shift the paradigm guiding the response to HIV epidemics, whilst negative results could challenge the case for continued investment in combination prevention interventions.

Despite being considered the gold standard for measuring the population-level impact of interventions, the design, implementation, and interpretation of C-RCTs can be extremely challenging [5],[8],[12],[13]. In the past, some researchers have turned to mathematical models once the studies were completed to help understand ambiguous and counter-intuitive results from C-RCTs [14]–[16]. Others have advocated for their use before studies begin to improve trial design [5],[17]–[21]. All three PEPFAR trials currently include an HIV transmission dynamic modelling component to complement traditional statistical approaches for the analysis of C-RCTs. Mathematical models will be used in three distinct phases—at the formative stage of trial planning, during the trial itself to monitor progress, and at the end of the trial to assist in interpretation and evaluation of short- and long-term impact.

In this review, we draw on results from a range of models to identify important considerations that should inform the design and interpretation of C-RCTs of combination interventions. We then propose how mathematical modelling can be integrated into the design, conduct, and analysis of the planned trials to complement traditional statistical approaches.

## Considerations for the Design, Conduct, and Interpretation of Cluster Randomized Controlled Trials

Previous modelling studies suggest that ART used alone or in combination with other interventions could significantly reduce long-term HIV transmission [4],[10],[22]–[26]. However, to evaluate the impact of interventions in the time frame of a trial, which is usually 2–3 y, it is critical to understand what magnitude of impact can be expected in the short term, whether the short-term impact is predictive of the long-term impact, and what implementation efforts might be required to achieve the desired level of impact. The answers to these questions are influenced by different determinants of the magnitude of intervention impact, and of the measurement and assessment of impact in C-RCTs. The important considerations and implications for C-RCTs for these determinants are summarised in Table 2. We provide illustrations of the main points below.

**Table 2:** Summary of important considerations for the design and interpretation of cluster randomized controlled trials (of combination interventions.

Important Considerations	Implications for Trials
**Determinants of the magnitude of intervention impact**	
Increase in intervention impact following the start of trial can be slow due to a number of delays before the full impact develops	Short-term impact will underestimate the long-term impact; substantially reducing HIV incidence over a trial of short duration will be challenging even with an ambitious combination intervention and rapid scale-up; it is important to set realistic expectations about the achievable magnitude of impact over the trial duration; this slow growth in impact can undermine the utility of stepped-wedge designs (with staggered randomized time of delivery of the intervention in each community) to measure a difference in HIV incidence between different interventions or components because the time interval between steps may need to be unfeasibly long^a^
The maximum impact of different intervention components is achieved at different times	The trial duration will influence which type of intervention seems to be the most effective; the overall impact of a combination intervention will be most strongly determined by different components at different times
The epidemiological context influences the intervention impact	The impact of the same intervention may not be the same across trials conducted in different epidemiological contexts; the results of the trial may not be generalisable to other settings
HIV prevalence and HIV incidence do not exhaustively describe the epidemiological context	This may introduce imbalance between the intervention and control arms, even after matching for HIV prevalence or even HIV incidence
The drivers of short-term and long-term impact can be different	Sufficient information on the epidemic drivers should be collected during the trial to help interpret trial results and to predict longer term impact
Distribution of coverage matters even at high coverage	Intervention impact can be substantially reduced if the intervention does not reach high-risk individuals; intervention impact can be substantially improved if the intervention does reach high transmitters; to understand trial results, detailed information on programmatic (e.g., coverage, uptake) and intermediate outcomes (e.g., change in behaviour, CD4 levels, viral load) by risk groups, age, and clinical status in both the intervention and control communities will be essential
**Challenges to the measurement of impact**	
Measurement of HIV incidence in a cohort over the whole trial duration, before the intervention has reached its full effect, underestimates the change in incidence that is achieved at the end of the trial	It would be better to measure incidence at the start and end of the trial using two independent cohorts with shorter follow-up
Evolving standard of care in control arm, as the coverage or scale-up of standard of care may improve over time	Reduces the contrast between intervention and control communities over time; our ability to measure a difference between trial arms will depend on the rapid scale-up of the intervention, having a large number of clusters to enable detection of smaller effects, or having trial duration longer than 2–3 y, to allow the intervention impact to be seen
Imbalance in key epidemiological characteristics between trial arms can occur, as HIV incidence and prevalence do not determine all key epidemiological characteristics that influence intervention impact	Could lead to a spurious indication that the intervention is working better or worse than it really did—matching clusters may be desirable; matching on HIV prevalence alone may not be sufficient, as trajectories in incidence and underlying patterns of risk behaviour across trial communities would not be captured
Dilution and contamination of the intervention impact may occur due to movement and sexual partnerships across multiple communities	The influence of the different sources of contamination on trial results will depend on the type of intervention; when there is extensive sexual contact between individuals from the trial arms, the measurable impact may be more strongly determined by acquisition-reducing than infectiousness-reducing interventions, such as ART; choosing distinct, independent communities will be important, especially to evaluate ART interventions

a Stepped-wedge design can still be useful for programme and intermediate outcomes, as changes in these outcomes can occur more rapidly than for HIV incidence or prevalence.

### Determinants of the Magnitude of Intervention Impact

#### Increase of intervention impact over time

**Figure 1:**
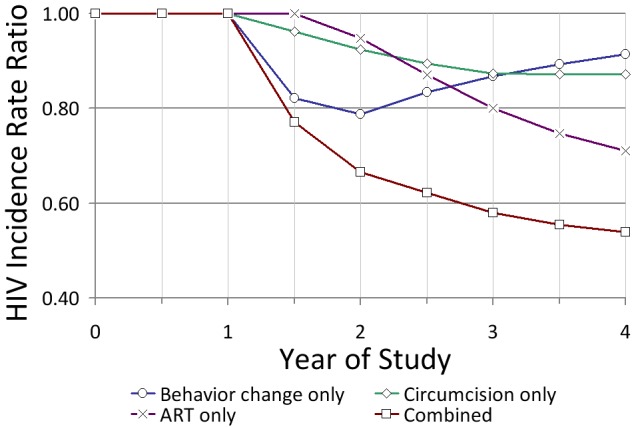
Predicted short-term impact of three intervention components linked to HIV testing in KwaZulu-Natal, South Africa. The model is based on a high-transmission setting under conditions of the current standard of care versus a high-coverage combination intervention (see [26]). The instantaneous HIV incidence rate ratio in the *y*-axis is intervention versus control. Impact estimates include an initial 6-mo period of preparation for the study. Assumptions for the combination intervention: 90% of adults in the intervention community are tested in the first year and thereafter every 4 y; those who test positive reduce risk behaviour for 3 y (on average) (25.0%/12.5% of men/women increase condom use; 25%/25% reduce partner acquisition); 70% of uncircumcised men are circumcised in the first year (efficacy = 60%); and all those in need of treatment (CD4 cell count <350 cells/µl) are immediately treated with ART (efficacy = 92%) with an annual dropout rate from treatment of 5%. The efficacy of MC in reducing susceptibility is assumed to be immediate (i.e., the wound healing period is negligible). Viral suppression for infected individuals once on treatment is immediate (i.e., no delay between treatment initiation and viral suppression). Assumptions for the standard of care: 20% of individuals test annually; 12.5%/6.5% of men/women who test positive increase condom use, and 12.5%/12.5% reduce partner acquisition, for one year; HIV-positive individuals are treated if CD4<200 cells/µl (dropout rate of 15%); and 27% of men are circumcised at baseline and 10% more over 4 y since the start of the intervention.

A concern of particular relevance for C-RCTs is that the full impact of interventions on HIV incidence at the population level is unlikely to be generated immediately after the start of the trial [16],[26]. For example, HIV risk might actually increase during the wound healing period following MC procedures [27]. In the case of ART, complete viral suppression and reduced infectivity takes time to occur after initiating treatment. Moreover, if ART eligibility is not immediate but occurs only once an individual reaches a predetermined CD4 level, as shown in Figure 1, there will be a lag between the start of the screening and treatment programme and the time point when the fraction of eligible HIV-positive individuals provided with ART is large enough to reduce transmission at the population level. This differs from I-RCTs, in which all eligible patients in the trial are immediately provided with their assigned treatment. In addition, in real-life situations, ART failure, poorer treatment adherence, and viral blips may be more frequent than in the ideal conditions of trials such as HPTN 052 [28], thereby reducing intervention impact. Finally, indirect benefits or “herd effects” accrued through the prevention of onward transmission, which are measurable in C-RCTs but not in I-RCTs, manifest more slowly, as these rely on a decreasing prevalence of HIV infections in some subpopulations [5]–[7].

Thus, C-RCTs designed to evaluate intervention impact after a short time will assess an impact that has not reached its maximum potential [16],[26]. For example, in Figure 1, HIV incidence is reduced by only 34% at 2 y even with a very ambitious combination intervention, compared with 66% after 25 y (not shown). Studies that estimate the intervention impact from changes in HIV prevalence, as is commonly done when monitoring key populations, have an even slower increase in intervention impact [13],[29]. Finally, because it can take different amounts of time for each intervention component to have its full effect, the overall impact of a combination intervention may be most strongly determined by different components at different time points after the start of the intervention programme (Figure 1) [26].

#### Influence of the epidemiological context

The epidemiological context for a given country or population is determined by the drivers of HIV transmission (e.g., patterns of risk behaviour and contact, and key biological factors that facilitate transmission) and by the past trajectory of the epidemic, which determines the distributions of individuals at different stages of HIV infection [30]–[36]. The underlying patterns and strength of transmission interact with the intervention and make predictions more complex. For example, for interventions that include expanded access to ART to prevent HIV (as will be the case in the three trials summarised in Table 1), the amount of transmission by an individual before treatment initiation, including during the initial highly infectious period, will determine the level of treatment required to reduce incidence. The amount of transmission generated early after infection depends on the number of concurrent sexual partners, the interval between sexual partnerships, the frequency and type of sexual acts, transmission probabilities, the fraction of new sexual partners who are already infected, and the prevalence of cofactors of HIV transmission, such as other sexually transmitted infections [36]–[39].

The effect of the same universal “test and treat” intervention can differ greatly across populations that have similar HIV prevalence, incidence, and rate of partner change but differences in other key sexual behaviours [31]. For instance, an intervention may reduce incidence by nearly 100% and eliminate the infection in one population if there is little heterogeneity in risk behaviour, whereas exactly the same intervention may reduce incidence by only 60% in another population if there is substantial heterogeneity and assortative mixing by sexual activity levels [31]. In a heterogeneous population transmission can persist within the highest risk group because individuals transmit rapidly after becoming infected and before getting ART. Thus, the impact of the same intervention may vary across C-RCTs conducted in different populations or settings, and, consequently, the findings from one trial may not necessarily apply to another setting. Mathematical models can take into account knowledge of the drivers of the HIV epidemic and the intervention impact in a specific trial setting, and help generalise trial results to other epidemiological contexts [5],[13],[21],[40].

#### Identifying drivers of short-term and long-term intervention impact

Although C-RCTs aim to measure the impact of interventions over a short period, broader public health interests are usually longer term. Factors that drive short-term impact may not be the same as those determining long-term impact and overall success of the programme. For example, one would expect the short-term impact of ART for prevention to be driven by factors such as the speed of linkage and retention in care during the first years after treatment initiation and adherence in the months following initiation, whereas long-term impact would be more sensitive to factors such as prolonged maintenance of retention in care and high adherence, continued frequent HIV testing, and robust linkage to care [22],[23],[26],[31]. Collection of data on these long-term factors may not be immediately useful for understanding the trial results in the short term, but will help predict the long-term impact of the trial results.

Finally, one important and often neglected consideration for C-RCTs is that most modelling analysis assumes that the intervention coverage is uniform with respect to different forms of risks and geography. This is unlikely to be the case in real world settings, as it is difficult to rollout an intervention with equal intensity in all settings, particularly if accessibility and outreach to key populations is poor [4],[22]–[26]. Modelling of a C-RCT of mass treatment of sexually transmitted diseases in Rakai, Uganda, showed that even if a high coverage is achieved overall, differential coverage in which those with highest sexual activity are not reached can severely attenuate the impact of the intervention [15]. Conversely, if those at highest risk can be effectively prioritised as coverage is increased, the impact of interventions can be enhanced [15],[32],[40]. Thus, collecting detailed information on programmatic, implementation, and intermediate outcomes (e.g., changes in behaviour, CD4 levels, and viral load) by risk group, age, and clinical status in both the intervention and control communities at different times during the trial is necessary for evaluation of the short-term and long-term impact.

### Challenges in Measuring Impact

Even if the intervention really does have an impact following rapid scale-up, high uptake, good adherence, etc., external factors may compromise our ability to measure a difference in impact between the intervention and control clusters.

#### Measuring HIV incidence over the whole trial duration

**Figure 2:**
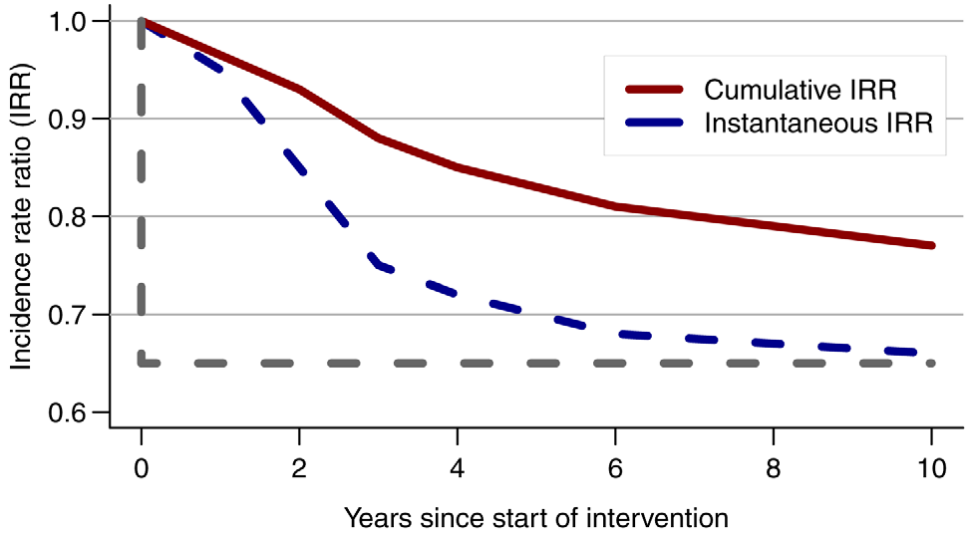
Consequence of measuring HIV incidence over the whole trial duration. Comparison of the instantaneous reduction in HIV incidence measured at one time point with the cumulative incidence rate ratio (IRR) measured over the whole trial duration (i.e., in a cohort that was initiated at the start of the trial) in a simulated population in Zimbabwe [16]. The grey dotted line shows the IRR if the full impact were achieved at the start of the intervention rather than after 10 y. The instantaneous IRR is 0.65 compared with only 0.77 for the cumulative IRR at year 10. From [16].

When incidence is measured in a single cohort over the whole duration of a trial, as currently planned in the three combination intervention trials (Table 1), the measured difference in incidence between the trial arms will be attenuated compared with the true difference that would be seen if HIV incidence were measured only at the end of the trial (Figure 2) [16]. This is because the measurement of incidence includes exposure while the intervention activities are still being ramped up and have not yet reached their full impact. Ideally, incidence should be measured at the start and end of the trial, using two independent samplings of the cohorts with shorter follow-ups. However, this solution may not be feasible in practice because of time constraints or costs. Thus, caution must be used when using modelling predictions of intervention impact based on predicted incidence at fixed time points (i.e., an instantaneous reduction in incidence) to estimate effect size and inform trial design.

#### Evolving standard of care in control arm

One of the strengths of C-RCT design is that it has a control group. One inescapable challenge, especially for the Johns Hopkins University/United States Agency for International Development (JHU/USAID) study, is that coverage with the standard of care in the control arm may increase over time, albeit more slowly than in the intervention arm, because of ongoing scaling-up activities for MC and/or changes in ART guidelines (from CD4<200 cells/µl to CD4<350 cells/µl). This can potentially reduce HIV incidence in the control arm and thereby reduce the contrast with the intervention arm, so compromising the power of the trial.

#### Imbalance between trial arms

One important and rarely acknowledged implication of the epidemiological context is that it could introduce an imbalance between trial arms, despite randomization and even if clusters are matched according to HIV incidence and/or prevalence. Such imbalance could lead to biases in either direction [8],[16]. Measurements of baseline HIV incidence before the start of the trial intervention, allowing the evaluation of “within cluster” changes in HIV incidence (before–after comparison), could help reduce this problem. However, this approach may not necessarily eliminate all confounding if differences in baseline HIV incidence actually reflect differences in key baseline epidemiological characteristics that influence how each community responds to interventions. Statistical adjustment limited to differences in cluster-level prevalence (or incidence) may only partially control for these nonlinear effects, especially if valid measures of most of the key potential confounding factors, and their interactions, are not available. Despite the benefit of randomization, which protects against known and unknown confounding, imbalance remains of particular concern in C-RCTs, as fewer units are randomized than in I-RCTs. For instance, there will be ∼24–30 clusters in the three planned C-RCTs versus ∼2,000 individuals in many I-RCTs [12].

Ideally, the number of clusters that are randomized needs to be sufficiently large to minimise the risk of imbalance or to allow matching of pairs or triplets of similar clusters, as proposed in the US Centers for Disease Control and Prevention/Harvard School of Public Health (CDC/HSPH) and PopART trials, using information on the epidemiological indicators available at the start of the trial. Whilst matching should help increase power if the matching indicators are highly correlated with the primary outcome [8], it can also be inefficient and reduce power if the matching indicators are not strongly related to outcomes. This could be the case when using only estimates of HIV prevalence. In addition, matching using several factors might not be feasible, as only a limited number of communities are available for most C-RCTs, and this might also limit the types of analyses that can be done [8],[41]. Due to limited information, especially on HIV prevalence at the cluster level in Iringa, Tanzania, a stratified approach is being adopted in the JHU/USAID trial.

#### Dilution and contamination

To minimise the risk of contamination, the clusters enrolled should be distinct, independent epidemiological units. Risk of contamination increases when individuals move or form sexual partnerships between clusters in different intervention arms of the trial or communities not enrolled in the trial. Individuals can also be lost to follow-up or can access an intervention not assigned to their cluster, thereby diluting the differences between arms.

The influence of the different sources of contamination on trial results will also vary for different interventions. For example, the impact of interventions that reduce acquisition of HIV, such as MC, should be only modestly affected by sexual mixing between communities, as long as residents in the intervention community are sufficiently exposed to the intervention. However, if substantial mixing occurs between communities, then interventions that reduce infectiousness such as ART may not have an observable impact in the intervention community. Choosing communities that are more isolated will therefore be more important for evaluating treatment as prevention than behaviour change communication or MC interventions. Although the risk of dilution and contamination can be minimised by choosing geographically separated communities, studies should still aim to collect data on sexual partnerships between communities; genetic sequencing technologies may be a useful for this [8],[42].

## The Role of Modelling in Planned and Future Cluster Randomized Controlled Trials

As discussed above, mathematical models have been useful to highlight important considerations relevant to C-RCTs. Based on this prior knowledge, we describe how mathematical models can be used before, during, and at the end of trials with reference to the three planned PEPFAR trials (Table 1), and with suggestions for future trials that may be planned subsequently.

### Modelling Prior to the Start of the Trial: Formative Phase

#### Informing design and intervention targets

Prior to the start of the trial, provided that sufficient data are available, models can be used to better understand the epidemic drivers in the trial communities and to define the combination intervention package most suited for the epidemiological context [40]. Then, models can be used, as in the three planned C-RCTs, to estimate the potential impact of the selected intervention in a given setting and to simulate how large a difference in HIV incidence (or prevalence, which is often used for key populations) will develop between the study arms over the trial duration, and how quickly it will develop. These impact estimates should take into account that the prevention activities occurring in the control arm may also evolve over the trial duration [13],[43]. Models can also be used to inform the minimum programmatic and implementation targets, such as the speed of scale-up and coverage of each intervention component, and/or the intermediate outcomes, such as change in behaviour, that are required to achieve the desirable impact or “effect size” at the end of the trial. Together, this information contributes to the overall design of the study.

Once a study design is chosen, models can also be used to simulate the process of the trial to identify potential difficulties such as the influence of sources of contamination or imbalance, to evaluate gain in power from matching clusters, or to validate sample size and power calculations [5],[16],[20]. All three C-RCTs are using models to simulate the influence of possible contamination. In addition, simulations can be used to control the chance of obtaining spurious significant results (type I error) when a novel design, such as an adaptive design that allows preplanned mid-course corrections, is used (see section on interim modelling analyses below) [5],[16]–[18],[20],[47]–[53].

#### Refinement of intervention package

Once calibrated to the specific trial setting using techniques previously described [13],[44]–[46], models can be used to refine the combination intervention package by assessing the impact of the different intervention components, such as promotion of condom use, MC, or ART, independently and in combination. This assessment can be achieved by varying the coverage, intensity, and uptake in different risk groups in the models. These modelling analyses help identify the minimum combined package (in terms of effort, persons reached, and resources spent) needed to maximise the short- and/or long-term impact, since the optimal package may depend on the time frame used to assess it [5],[26],[27],[32],[33]. These analyses can provide useful information about the attenuation of impact that could ensue from worse coverage in populations at the highest risk of infection, or from scaling up one component more quickly than another.

### Modelling during the Trial: Interim Modelling Analyses

Although statistical methods for formal interim efficacy review of phase III I-RCTs can theoretically be adapted for monitoring C-RCTs [52], they may be logistically more challenging, especially for short C-RCT trials, if HIV incidence measurements are required soon after the start of the trial. We propose the innovative use of mathematical modelling to conduct interim analyses, when interim HIV incidence data are not available, to allow the ongoing trials to be modified or adapted to reduce the likelihood of inconclusive outcomes.

The planned C-RCTs commissioned by PEPFAR are particularly ambitious, aiming to reduce HIV incidence by 25%–60% in just 2 or 3 y (Table 1). As currently proposed by the JHU/USAID team, mathematical modelling can be used to help monitor the progress of the trial. This can help assess the quality of the implementation and, if needed, trigger predetermined mid-course corrections as part of an adaptive design, such as accelerated roll-out or modified trial duration [48]–[51]. For example, a minimum level of coverage (at specific time points) under which the trial will probably be unsuccessful can be predetermined. In addition, interim modelling analyses can be done using additional data from the baseline surveys in each trial cluster (such as sexual behaviour and updated HIV prevalence estimates) and the most recent information on process indicators of coverage and intensity that is available. Robust monitoring and evaluation data will be necessary to permit these kinds of analyses in a timely fashion. The objective is to predict the likely impact at the end of trial and to estimate the probability that an effect size will be detected. This is similar to a conditional power analysis for futility stopping conducted at the interim analysis of an I-RCT, after which the trial is stopped if the interim results suggest that the effect sought is unlikely to be achieved if the trial continues. This approach is particularly relevant in situations in which no interim incidence measurements are available to conduct a formal interim analysis.

**Figure 3:**
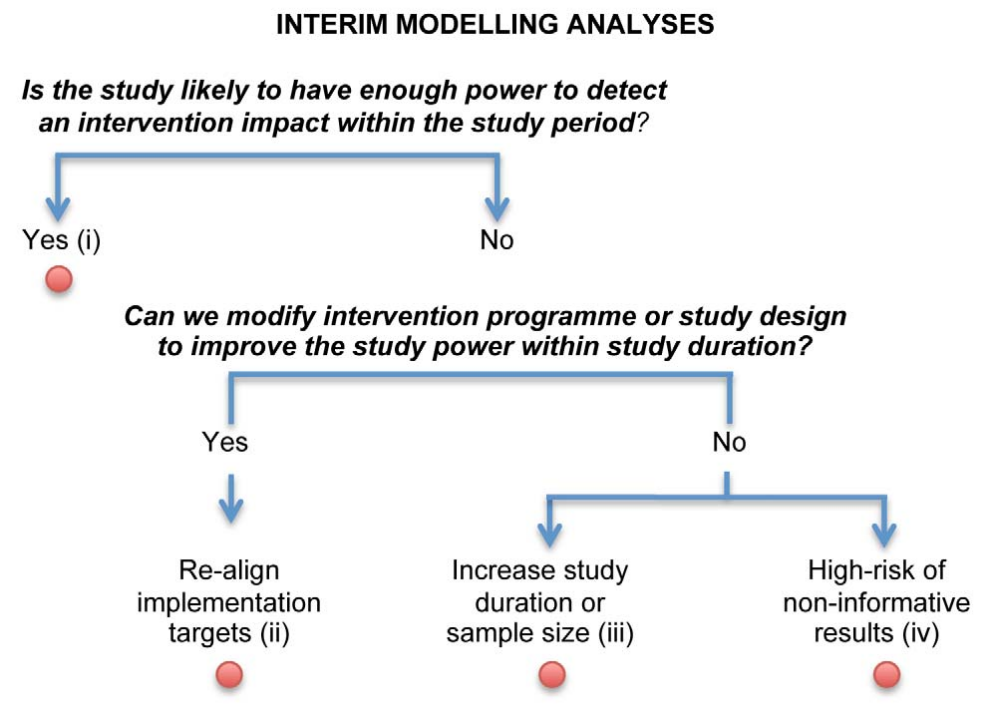
Logical flow of interim modelling analyses. This approach uses available data from the baseline surveys in each trial cluster and information on process indicators of coverage and intensity available for each cluster within each trial arm gathered after the start of the trial. These data would not include observed HIV incidence. The interim modelling analysis may come to one of four conclusions. (i) The targeted effect size on HIV is likely to be achieved at the end of the study without having to modify the intervention targets/implementation strategy. (ii) The targeted effect size is unlikely to be achieved, and therefore the intervention targets/implementation strategy need to be revised. (iii) The targeted effect size is unlikely to be achieved, even if the intervention targets are improved to their realistic maximum, unless there is a change in the study design (such as an increase in sample size or study duration). (iv) There is little chance of being able to detect an impact at the end of the trial even if the study duration is increased. The number of interim analyses should be predetermined at the start of the trial and take into account trial characteristics, logistical considerations (such as the time and cost required to regularly update programmatic data during the trial and to perform the modelling analyses), and the statistical effect of the interim analysis and proposed changes on the overall type I error.

The information gained from this type of modelling can then be used to guide the conduct of the rest of the trial (Figure 3). The question of particular interest is to determine, with the level of coverage and intensity achieved between baseline and interim analysis, the likelihood of observing a measurable impact at the end of the trial and whether changes to the implementation of the intervention or conduct of the study are required to maximise its usefulness. When considering allowing modifications of some prespecified aspect of the design based on interim analysis, it is necessary to consider its possible influence on the overall type I error (chance of detecting a false positive result). Although the type I error is usually well controlled with traditional (non-adaptive) trial designs, this is generally not the case for adaptive trial designs, where inflation of the type I error is often a concern [48],[49],[53]. Thus, mid-course corrections should be carefully planned and implemented using trial simulations to demonstrate that the type I error will be protected [49]–[51],[53]. The interim modelling analysis may come to one of the four conclusions shown in Figure 3. For example, a finding that there is little chance of detecting an impact even if the study lasted longer (outcome iv) would indicate a high likelihood of obtaining non-informative results, akin to the concept of “futility” in I-RCTs.

This information should be used as a warning of potential problems, and the recommended action might include improving programmatic targets with or without increasing study duration. Those decisions should be discussed within the framework of the independent data monitoring committee that oversees the conduct of the trial, the quality of the implementation, and impact projection. The data monitoring committee could endorse the trial protocol team's decision and/or recommend modifications of the trial. At least one or two members of the data monitoring committee should have expertise in mathematical modelling.

### Modelling at the End of the Trial: Evaluation, Interpretation, and Extrapolation

**Figure 4:**
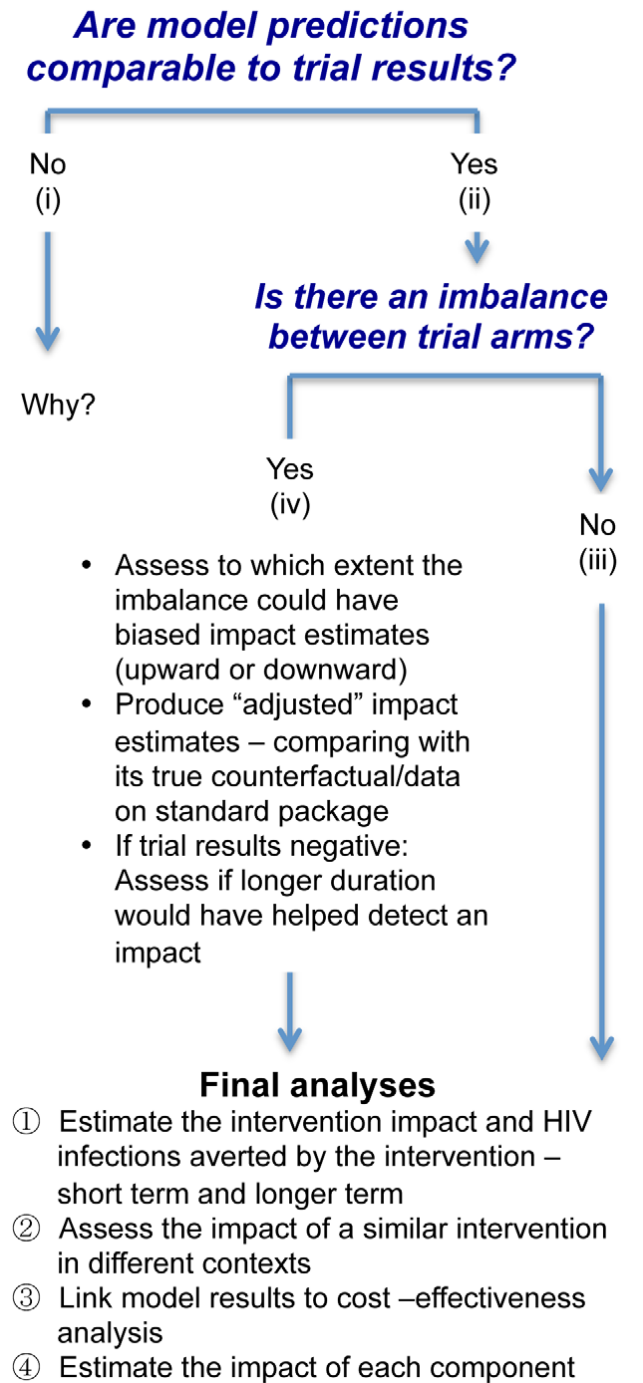
Logical flow of modelling stages for the final impact analyses.

Depending on the outcome of the trial, models can be used in slightly different ways to help interpret the trial results (Figure 4) [5],[13]–[16],[29],[31],[43],[44]. The first goal of this final set of analyses is to test and potentially validate final model predictions of intervention impact at the end of the trial. To do this, the analyses should use all the relevant available data on sexual behaviour as well as process indicators of intervention coverage and intensity collected in each community and trial arm during the whole trial duration, to inform prior model parameter distribution and calibrate the model to the HIV outcomes. For validation purposes, model predictions should ideally be derived just before the end of the trial, while the modeller is still blind to the empirical trial results on HIV incidence.

If the model predictions and trial results are similar, then this validates the model projections, and the model can be used for further analyses with a greater degree of confidence. If not, refinements in the statistical analysis, such as adjustment for baseline factors, and/or in the mathematical model are required until the source of the discrepancies is identified, as shown in Figure 4.

If the trial results suggest that the intervention has a significant impact and there is no imbalance in key indicators of epidemiological context between the trial arms, the final modelling analysis can predict the number of infections that would be averted by the combined package in the intervention arm compared with the standard package in the control arm over different time periods if the intervention were continued. The counterfactual would be simulated using the level of coverage and change in behaviour and other programmatic outcomes observed with the standard package in the control arm over the course of the study. The models could also be adapted to project impact in other populations with different epidemiological contexts to help generalise the trial results, and to compare the results with those of other trials of combination interventions. Provided that good costing data are also collected (as is planned in all three trials), it will be possible to link the costing data to the short- and long-term model predictions for a cost-effectiveness analysis [54]. One of the challenges will be to understand the costs incurred for the intervention in the trial, including start-up, small-scale set-up, and cost of the learning curve, compared with how these costs would evolve in a large-scale programme over the long term [54].

Currently, C-RCTs are not designed to establish differences between the different intervention components, as this would require larger trials with multiple arms, potentially using factorial designs. It may be possible to model and predict the impact of each specific component of the intervention package independently, but it will be challenging. If individuals were exposed to several intervention components during the trial, it would be difficult to attribute an observed reduction in risk behaviour, e.g., relating to sexual behaviour or adherence, to one particular component. Also, with the acknowledged limitations of the collection of behavioural data, it is difficult to reliably transduce the effects of reported changes in behaviour into an impact on transmission. It may be more feasible to link interventions that have hard end points, such as being circumcised or starting on ART, to estimated impact. The epidemiological synergy between interventions, which can make the impact of combination prevention greater than the sum (or multiplication) of its parts, may also be an important part of the total impact. Conversely, redundancy between components may reduce the combined intervention impact, meaning that the total intervention impact may be lower than the sum (or multiplication) of its parts.

If there is a significant imbalance in key baseline characteristics between trial arms, it would be useful to assess the extent to which this imbalance could have biased the observed impact estimate, and to produce “adjusted” estimates, i.e., estimates revised upward in the case of a positive trial or downward in the case of a negative trial.

Finally, if a trial produces negative results despite the coverage of interventions such as ART and MC increasing substantially, the main points to explore would be the following: to what extent this lack of impact was because the trial was too short; how long would it have taken to detect a measurable impact; and whether the level of contamination in the control group was too high.

## The Way Forward

In this exciting new era of HIV prevention technologies, C-RCTs will be used to test the hypothesis that combination HIV prevention, including expanded access to ART, can substantially reduce HIV incidence. Of particular relevance for the three planned C-RCTs is the observation that it may be challenging to observe a substantial reduction in HIV incidence (>40% reduction) over the 2- to 3-y duration of a trial unless the interventions are scaled up rapidly and the key populations are reached quickly. Models that reflect realistic delays in implementation and scale-up, as well as delays in the development of direct and indirect effects calibrated to the specific trial settings, will be particularly useful. These models will provide estimates of the effect size that can be expected at the end of the trial, the programmatic and implementation targets required to generate this effect, and the projected long-term impact. Ideally, the effect size should be chosen to be of public health relevance and to reflect long-term goals [5].

Given the challenges in scaling up interventions rapidly and the importance of these current trials, interim modelling analysis can provide a very useful and innovative tool to project the final intervention impact and to adopt mid-course corrections to accelerate scale-up and minimise the chance of having inconclusive trial results. However, the adaptive features of this design require careful statistical considerations so not to inflate the false positive rate, which in turn requires modelling analysis to determine when that risk is outweighed by potential benefits.

The proposed modelling analyses will require collection of detailed data prior to and during the trial about the epidemiological context, and detailed information about the programmatic outcomes of each component will need to be available in a timely manner for key populations. Thus, it is critical that efficient data-capture systems are in place to allow linkage of HIV testing to the different services and the other components being modelled. There is also an emerging consensus that collecting detailed data characterising sexual networks will be important to interpret the results of the different trials effectively, especially if negative results are obtained. Efforts are currently ongoing to harmonise survey instruments across settings. The feasibility and added value of conducting complementary phylogenetic analyses to help understand transmission networks is also being considered.

Importantly, the interactive use of mathematical models during C-RCTs in a carefully preplanned fashion will not only be useful to demonstrate the use of models in designing, conducting, and interpreting C-RCTs, but will also provide a unique opportunity to validate and refine model projections. It will also test the usefulness of this modelling framework, which could then be used for C-RCTs designed to test prevention interventions for other infectious diseases with complex transmission dynamics such as malaria, tuberculosis, and neglected tropical infections.

**Key Points**

- Cluster randomized controlled trials (C-RCTs) are currently planned to evaluate whether combination HIV prevention, including expanded access to ART, can substantially reduce HIV incidence at the population level in southern and eastern Africa.
- It may be challenging to observe a substantial reduction in HIV incidence (>40% reduction) over the 2- to 3-y duration of a C-RCT, unless the interventions are scaled up rapidly and the key populations are reached quickly.
- Mathematical models can and will be used to complement C-RCTs before, during, and after their completion to help plan, conduct, and interpret trial results and strengthen the evaluation of these interventions.
- Given the challenges in scaling up interventions rapidly and the importance of these current trials, we propose the innovative use of mathematical modelling to conduct interim analyses to modify or adapt an ongoing trial (in a carefully planned and prespecified manner) to reduce the likelihood of inconclusive trial outcomes, when interim HIV incidence data are not available.
- The interactive use of mathematical models during C-RCTs in a carefully preplanned fashion will also provide a unique opportunity to validate and refine model projections.

## Abbreviations

ART: antiretroviral therapy
C-RCT: cluster randomized controlled trial
I-RCT: individual-based randomized controlled trial
JHU/USAID: Johns Hopkins University/United States Agency for International Development
MC: male circumcision
PEPFAR: US President's Emergency Plan for AIDS Relief
